# Impact of prior underinsurance on cervical cancer screening among Davidson County, Tennessee, women diagnosed with invasive cervical cancer, 2008–2018

**DOI:** 10.1186/s12905-022-01638-9

**Published:** 2022-03-12

**Authors:** Emmanuel N. S. Sackey, Manideepthi Pemmaraju, Marie R. Griffin, Jessica L. Castilho

**Affiliations:** 1 Department of Health Policy, Vanderbilt University Medical Center, Nashville, TN USA; 2 Division of Infectious Diseases, Department of Medicine, Vanderbilt University Medical Center, Nashville, TN USA

**Keywords:** Cervical cancer, Insurance, Human papillomavirus (HPV), Surveillance

## Abstract

**Introduction:**

We sought to investigate the association between insurance coverage history and cervical cancer screening among Davidson County, Tennessee, women diagnosed with incident cervical cancer.

**Methods:**

We reviewed medical records of women diagnosed with invasive cervical cancer from 2008 through 2018 identified via the state's cancer registry and by active surveillance of diagnostic pathology reports for the HPV-IMPACT project. Per 2012 United States Preventive Services Task Force recommended cervical cancer screening guidelines, women were characterized into three screening history categories: “no screening”, “no follow-up” and “test/screening failure”. Multivariable logistic regression measured the association of prior inadequate insurance (underinsurance) and screening history (“no screening/no follow-up” compared to “test/screening failure”).

**Results:**

Of 212 women, most (77%) had not undergone recommended cervical cancer screening or follow-up prior to cancer diagnosis. Overall, 28% of women had history of underinsurance in 5 years prior to diagnosis. In adjusted analyses, underinsured women were more likely to have a “no screening/no follow-up” prior to cancer diagnosis (aOR 4.26; 95% CI 1.15–15.80) compared to “test/screening failure” history. Non-white race (aOR 2.73; 95% CI 0.98–7.61), older age (aOR 1.03 per year; 95% CI 1.00–1.07), and history of smoking (aOR 4.07; 95% CI 1.54–10.74) were also associated with increased likelihood of “no screening/no follow-up”.

**Conclusions:**

Previous underinsurance was independently associated with non-adherence to cervical cancer screening and follow-up guidelines among women with incident cervical cancer. Further study of factors contributing to inadequate cervical cancer screening and interventions to increase cervical cancer screening in high-risk populations is needed.

**Supplementary Information:**

The online version contains supplementary material available at 10.1186/s12905-022-01638-9.

## Introduction

The significant reduction in cervical cancer disease burden in the United States (US) has largely been attributed to cervical screening programs [1, 2]. Despite these gains, US cervical cancer incidence rates have stagnated over the past decade, with 8.2 and 7.7 cases per 100,000 women recorded in 2006 and 2016, respectively [3, 4]. The Southern US, and especially Tennessee (TN), is disproportionately affected by higher disease burden (reported average age-adjusted annual incidence of 8.4 per 100,000; 2013–2017 in TN) along with lower cervical cancer screening rates compared to the national average, highlighting one of several disease burden disparities observed within the US [4–7].

Lack of screening and appropriate follow-up (i.e. non-adherence to recommended guidelines) are the most common clinical scenarios preceding an invasive cervical cancer diagnosis [8, 9]. Recommended cervical cancer screening guidelines updated in 2012, introduced longer screening intervals and incorporation of molecular human papillomavirus (HPV) testing for a more robust secondary prevention strategy [1, 10, 11]. Less frequent screening allows time for HPV infections to resolve, minimizing the frequency of invasive management of lesions likely to spontaneously regress while maintaining a low likelihood of missing progression from precancerous cervical intraepithelial neoplasia (CIN) to invasive cervical cancer [12]. These guideline changes have resulted in intended declines in annual screening rates across all age groups and increased use of molecular HPV testing (either alone or as part of co-testing) [1, 13, 14]. However, overall screening adherence rates across all screening-eligible age groups in the US remain below intended targets [1, 13].

Social determinants of health (social factors besides medical care that impact individual and population health indices) including barriers to health care access and insurance coverage have been shown to affect cervical cancer screening rates, cervical cancer burden, and disease attributes [15]. Although introduction of the Affordable Care Act (ACA) in 2010, resulted in great declines in the uninsured rates among non-elderly Americans—reaching an all-time low in 2016—uninsured rates have begun to steadily rise in recent years. Individual states’ decisions concerning Medicaid expansion adoption under the ACA—TN being a non-expansion state—have heightened concerns regarding the impact of insurance issues on disparities in preventive health services such as cancer screening [16–18]. Davidson County, TN, a rapidly growing, diverse population reported higher uninsured rates (17.8%) in 2017 than the state (15.9%) and the country (14.8%) along with worsening related social determinants health which are likely to affect the health-seeking behaviors of residents [19, 20].

Few studies have examined how prior insurance coverage affects cervical cancer screening among women in a defined population with incident cervical cancer. Prior studies have assessed either associations between insurance and screening rates in screening-eligible populations or predictors of cervical screening or invasive cancer [1–3, 13, 21]. However, these studies have been limited to select populations, such as insured women enrolled in a single healthcare network. Population-level analyses of women in the US with cervical cancer are limited by the absence of important clinical and social data available through cancer registries. In a recent study, women with invasive cervical cancer identified through cancer registries from three US states (2013–2016), utilized supplemental medical record review and survey data collection to show that women who received screening before their cancer diagnosis were more likely to be of a younger age, have higher income and have insurance. However, that study was limited to women who agreed to participate and provide survey responses [22]. In this study, we analyzed the association between a prior lack of or insufficient insurance coverage and cervical cancer screening histories among women in Davidson County diagnosed with invasive cervical cancer from 2008 through 2018. We hypothesized that women with a history of no insurance or underinsurance would be more likely to miss screening or have inadequate follow-up after abnormal screening test results compared to women with a history of consistent insurance coverage.

## Methods

### Data sources and population

The Human Papillomavirus Vaccine Impact Monitoring Project (HPV-IMPACT) is a multi-site, US population-based, active cervical precancer surveillance project funded by the Centers for Disease Control and Prevention (CDC) [23]. Beginning in 2019, the project expanded its activities to include invasive cervical cancer surveillance retrospectively from 2008. We used data collected by the TN HPV-IMPACT site (catchment area: Davidson County) that uses diagnostic pathology laboratory reports to identify high-grade cervical pre-cancerous lesions i.e., cervical intraepithelial neoplasia grades 2 and higher and adenocarcinoma in situ*,* collectively referred to as CIN2+ and invasive cervical cancer cases to assess population-level effectiveness of HPV vaccination [24].

We identified female residents of Davidson County, TN, diagnosed with invasive cervical cancer between January 1, 2008, and December 31, 2018, aged ≥ 18 years at the time of diagnosis. The total population of women aged ≥ 18 years in this catchment area was 305,982 in 2010 [25]. All invasive cervical cancer cases were identified through: (1) diagnoses reported to the TN Cancer Registry and (2) comprehensive review of laboratory reported pathology records. We excluded all women who had a cervical carcinoma in-situ (i.e., non-invasive carcinoma) diagnosis, were living out of the catchment area at the time of diagnosis or had a non-cervical primary tumor.

This project was considered public health surveillance (i.e., not human research) by Institutional Review Boards at Vanderbilt University Medical Center, TN Department of Health, and CDC and received a non-Research determination under 45 CFR 164.512.

### Data collection

Following identification of all potential cases within the study period from the cancer registry and partnering laboratories, trained study personnel systematically abstracted socio-demographic and cancer diagnosis information from detailed medical records review using standardized case report forms. Despite our data identification sources providing some socio-demographic and other cancer-related information, we abstracted and validated all data elements for this study through medical records review.

Date of cancer diagnosis was determined as the first date on which the specimen obtained from a surgical procedure (including cervical biopsies/excisions, endocervical curettage, or hysterectomy) indicated invasive cervical cancer. Cancers diagnosed on cervical cytology only were excluded. We collected the following at the time of cancer diagnosis: race/ethnicity (non-Hispanic White, non-Hispanic Black, Hispanic and other/unknown), address, insurance status (private, public, none, unknown), cancer stage (International Federation of Obstetrics and Gynecology [FIGO] staging criteria), any symptoms reported during diagnostic work-up, smoking status, and any immunocompromising conditions (complete list of conditions in Additional file 1: Table S1). We abstracted all available data on previous cervical cancer screening (including cytology and molecular HPV tests), follow-up exams, and cervical procedures up to 5.5 years prior to the cervical cancer diagnosis. To identify potential contributory barriers to screening, we collected documentation of any of the following in the five years prior to cancer diagnosis (indicated as “yes”, “no” or “unknown”) from the medical records. Underinsurance, our primary exposure variable, was defined as any documented history of no insurance or insufficient coverage, including lapses in coverage or concerns about poor insurance coverage resulting in lack of or delayed receipt of healthcare services. This information was searched for and collected in multiple ways which included: (1) provider notes stating a lack of or gaps in insurance coverage affecting screening or healthcare seeking behavior; (2) review of insurance information provided in “sign-in” or “face sheet” information for all available prior clinic encounters for any documentation of being uninsured or underinsured (e.g., charity; indigent; assistance from the Tennessee Breast and Cervical Screening program). Other potential barriers to screening noted included documentation of poor English language proficiency, addiction/substance use disorder, morbid obesity or documented body mass index > 40 kg/m^2^, history of homelessness, history of incarceration, and diagnosis of a serious mental health disorder (using Substance Abuse and Mental Health Services Administration definition) [26].

The medical record review involved a comprehensive approach to obtain all available records for data abstraction. Our data identification sources (i.e., cancer registry and pathology laboratories) provided information on the ordering provider of the qualifying diagnostic procedure and associated provider facility. With this information, medical records were obtained either through electronic health records systems or paper records upon request from facilities. Missing information following this initial review was sought by obtaining records from gynecology oncology providers or primary care physicians/obstetricians-gynecologists women had been referred to initially or routinely visited as part of their gynecological care respectively. This multi-step approach meant patient records were examined from at least two to three different providers per woman and as a thorough investigation of the continuum of care for each woman as possible.

### Defining cervical cancer screening history

In determining cervical cancer screening history, we excluded all cervical cytology and HPV tests conducted in the six-month period prior to cancer diagnosis as we assumed these to be part of the diagnostic process and not as part of routine cervical cancer screening. We used a six-month “cut-off” as has been used in prior studies [8, 22]. We then classified women into one of three mutually exclusive screening history categories based on the documentation of a screening test performed as per 2012 United States Preventive Services Task Force (USPSTF) recommended screening intervals: six months—3.5 years for a cytology test only and six months—5.5 years for cytology with HPV molecular test or HPV molecular test only [27]. We assumed that adherence to the 2012 guidelines would prevent progression to cervical cancer for most women. The "no screening" category included women who had no history of a screening test (cytology, HPV, or cytology/HPV co-test) within the recommended screening interval immediately prior to cancer diagnosis. Women in the "no follow-up" group had at least, either a ≥ six-month or ≥ 1 year lapse between abnormal cytology and/or positive HPV test and receipt of recommended follow-up management depending on the grade and severity of the abnormal screening test, as per American Society for Colposcopy and Cervical Pathology (ASCCP) guidelines [28]. Finally, women whose most recent screening test in the recommended timing interval prior to cancer diagnosis was a normal cytology test and/or a negative HPV molecular test or women adequately followed up after an abnormal screening test as per ASCCP guidelines were classified as "test/screening failure”. Women with no or inconsistent information on screening tests performed in the recommended screening interval were considered to have an “indeterminate” screening history and were excluded from analyses examining patient characteristics by screening history. An algorithm of the screening history determination process is shown in Additional file 1: Figure S1.

### Statistical analysis

For women with an invasive cervical cancer diagnosis, we summarized demographic, social, and clinical characteristics by the three screening history categories and examined the association between screening history and women’s characteristics using parametric and non-parametric tests (Pearson’s chi-square [two-sided]; Fisher’s exact; Wilcoxon rank sum; Kruskal–Wallis) as applicable. Using similar tests, we examined the association between underinsurance (underinsurance versus no such history) and women’s sociodemographic and clinical characteristics. Univariate and multivariable logistic regression models were used to test our hypothesis that women with a history of underinsurance in the 5-year period leading up to cancer diagnosis were at an increased odds of having the composite “no screening/no follow-up” screening history versus “test/screening failure” compared to women with no history of underinsurance. The covariates included in the multivariable logistic regression were determined a priori based on pre-existing knowledge of their effects on the exposure (insurance history) and outcome (screening history) variables. We adjusted for race/ethnicity, cancer stage, smoking status, presence of one or more barriers to screening other than underinsurance and year of cancer diagnosis. All analyses were conducted using STATA (version 16) with statistical significance determined at α = 0.05. All statistical tests were two-sided.

## Results

**Fig. 1:**
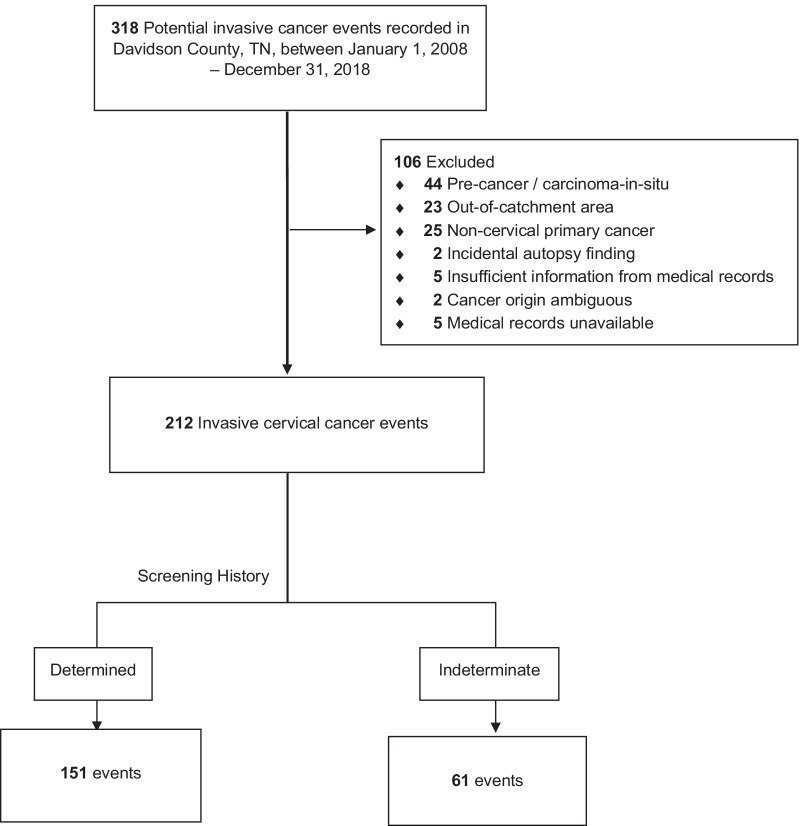
Study population

We reviewed the medical records of 318 potential invasive cervical cancer events within the study period received from our data sources. There were 106 (33%) events classified as ineligible due to being out of catchment area, precancer diagnosis, noncervical primary malignancy, incidental autopsy findings, incomplete or unavailable medical records or ambiguity in origin of cancer diagnosis, leaving 212 (66%) events that met the study’s eligibility criteria. (Fig. 1).

We summarized the demographic and clinical characteristics of women with cancer by any documented history of underinsurance coverage in Additional file 1: Table S2. Of all 212 women with validated cancer events, 60 (28%) had a history of underinsurance in the 5 years prior to diagnosis including 38 (18%) with no documented public or private insurance at the time of the diagnostic procedure. Overall, the median age at cancer diagnosis was 50 years and 31 (15%) women were ≥ 65 years of age. Seventy-four (35%) cancers were of non-squamous histology (28% adenocarcinoma; 2% adenosquamous; 5% other carcinomas, including small cell, adenosarcoma, large cell neuroendocrine, lymphoepithelial-like and adenoid cystic carcinomas of the cervix. Eighty percent of women (n = 164) presented with symptoms at diagnosis, with 91% (149 of 164) of these reporting vaginal bleeding. The median age and year at diagnosis, cancer stage, presence of any immunocompromising condition and smoking status did not differ significantly between underinsured women and those with no such history. Within race/ethnicity groups, Hispanic women recorded the greatest percentage (55%; 11 of 20) of underinsurance. More women with a history of underinsurance compared to women without presented with symptoms (93% versus 75%). Women with prior underinsurance were more likely to have one or more of the additional potential screening barriers (“other barriers”) in the five-year period prior to invasive cancer diagnosis, the most frequent of which was poor English proficiency (documented in 23% of women with prior underinsurance compared to only 7% of women without underinsurance). Other potential barriers to cervical cancer screening were rare and documented in less than 10% of women overall (not shown in the table).

Of the 212 validated invasive cancer events, 151 (71%) had sufficient information in their medical records to determine their screening histories (Fig. 1). These 151 women represented the denominator for analyses by screening history categories and the study hypothesis. Women excluded from analyses involving screening history due missing information on prior screening tests (“indeterminate screening history”) had broadly similar characteristics to those included. One notable significant difference was that women with indeterminate screening histories were diagnosed a median 2 years earlier, highlighting challenges in retrieving relevant information from medical records of women diagnosed in earlier years (characteristics are summarized in Additional file 1: Table S3). Table 1 shows the demographic and clinical characteristics of the 151 women whose screening history could be determined. In total, 94 (62%) were classified in the “no screening” category, 22 (15%) as “no follow-up,” and 35 (23%) as “test/screening failure”. The median age, presence of symptoms, history of smoking, cancer stage and histology type (squamous, non-squamous, unknown) varied significantly across the screening history categories.

**Table 1:** Patient characteristics by screening history among Davidson County, TN, women diagnosed with invasive cervical cancer: 2008–2018

Characteristic	No screening (N = 94)	No follow-up (N = 22)	Test/screening failure (N = 35)	Total (N = 151)	*p* value^ǂ^
Median age at diagnosis [IQR]	53 [44–62]	46 [36–55]	44 [34–58]	51 [40–59]	0.007**
Median year of diagnosis	2013	2014	2013	2013	0.898** ± **
[IQR]	[2011–2016]	[2011–2016]	[2010–2016]	[2011–2016]	
Race/Ethnicity					
White, non-Hispanic	53 (56)	14 (64)	27 (77)	94 (62)	0.527
Black, non-Hispanic	26 (28)	6 (27)	6 (17)	38 (25)	
Hispanic	11 (12)	2 (9)	2 (6)	15 (10)	
Other	4 (4)	0 (0)	0 (0)	4 (3)	
Symptoms at diagnosis					
Yes	81 (86)	15 (69)	21 (60)	117 (78)	0.004**
No/unknown	13 (14)	7 (32)	14 (40)	34 (22)	
Immunocompromised					
Yes	5 (5)	1 (5)	1 (3)	7 (5)	1.000
No/unknown	89 (95)	21 (95)	29 (97)	144 (95)	
Current/Past smoker					
Yes	45 (48)	16 (73)	10 (29)	71 (47)	0.005**
No/unknown	49 (52)	6 (27)	25 (71)	80 (53)	
Histology type					
Squamous	61 (65)	18 (82)	15 (43)	94 (62)	0.019*
Non-squamous	31 (33)	4 (18)	20 (57)	55 (36)	
Unknown	2 (2)	0 (0)	0 (0)	2 (1)	
Stage (FIGO)					
Stage I-IIA (local)	45 (48)	16 (73)	25 (71)	86 (57)	0.011*
Stage IIB-IV (advanced)	47 (50)	5 (23)	8 (23)	60 (40)	
Unknown/missing	2 (2)	1 (4)	2 (6)	5 (3)	
History of underinsurance^a^					
Yes	36 (38)	7 (32)	4 (11)	47 (31)	0.014*
No/unknown	58 (62)	15 (68)	31 (89)	104 (69)	
Other barriers^b^					
None	60 (64)	12 (55)	26 (74)	98 (65)	0.296
1 or more barriers	34 (36)	10 (45)	9 (26)	53 (35)	

^**ǂ**^Pearson’s chi-square/Fisher’s exact tests as applicable

^**±**^Wilcoxon rank-sum test

() Column percentages

*IQR* interquartile range

Boldface *p* value indicates statistical significance (*p < 0.05, **p < 0.01, ***p < 0.001)

^a^Underinsurance defined as any history of no insurance or insufficient coverage, including lapses in coverage or concerns about poor insurance coverage resulting in lack or delayed receipt of healthcare services

^b^Other barriers includes documentation of any of the following in the five years prior to cancer diagnosis: poor English proficiency, substance use disorder, morbid obesity (body mass index ≥ 40 kg/m^2^), history of incarceration, history of homelessness, or serious mental illness

Underinsured women had a greater odd of having a “no screening/no follow-up” screening history compared to women with no documented history of underinsurance, as shown in Table 2. After adjusting for age, race/ethnicity, smoking status, cancer stage, documentation of other potential screening barriers, and year of diagnosis, a history of underinsurance significantly increased a woman’s odds of having a “no screening/no follow-up” screening history (adjusted odds ratio = 4.26, 95% CI: 1.15–15.80) versus test/screening failure. Women of older age, women of non-white race or Hispanic ethnicity, and women with a history of smoking were also at increased odds of having “no screening/no follow-up” histories, although median age at diagnosis and race/ethnicity did not quite meet statistical significance.

**Table 2:** Univariate and Multivariable Logistic Regression (No screening/follow-up vs test/screening failure)^a^

	No screening/follow-up (N = 116)	Test/screening failure (N = 35)	Unadjusted odds ratio (95% CI)	Adjusted odds ratio (95% CI)^a^
History of underinsurance				
No	73 (63)	31 (89)	1.00 (ref.)	1.00 (ref.)
Yes	43 (37)	4 (11)	**4.57 (1.51–13.82)**	**4.26 (1.15–15.80)**
Median age at diagnosis	52	35	1.04 (1.01–1.07)	1.03 (1.00–1.07)
[IQR]	[43–62]	[34–58]		
Race/Ethnicity				
White, non-Hispanic	66 (58)	27 (77)	1.00 (ref)	1.00 (ref)
Non-white or Hispanic	48 (42)	8 (23)	2.47 (1.03–5.90)	2.73 (0.98–7.61)
FIGO stage				
I-IIA	58 (53)	25 (79)	1.00 (ref)	1.00 (ref)
IIB-IV	51 (45)	8 (21)	2.66 (1.11–6.41)	2.17 (0.82–5.78)
Unknown/missing	3 (3)	2 (6)	–	–
Current/past smoker				
No/unknown	55 (47)	25 (71)	1.00 (ref.)	1.00 (ref.)
Yes	61 (53)	10 (29)	**2.77 (1.22–6.29)**	**4.07 (1.54–10.74)**
Other barriers^b^				
None	72 (62)	26 (74)	1.00 (ref.)	1.00 (ref.)
1 or more	44 (38)	9 (26)	1.76 (0.75–4.11)	1.48 (0.54–4.07)
Median year of diagnosis [IQR]	2013	2013	1.01 (0.89–1.14)	1.00 (0.87–1.16)
	[2011–2016]	[2010–2016]		

() Column percentages

*IQR* interquartile range

Boldface odds ratio value indicates statistical significance

^a^Adjusted for age (as a continuous variable), race/ethnicity, smoking status, stage, presence of one or more barriers to screening other than underinsurance and year of cancer diagnosis (as a continuous variable)

^b^All other barriers to cervical cancer screening other than underinsurance

## Discussion

Among women with incident cervical cancer and available screening history data, the overwhelming majority of women (77%) had not received guideline recommended preventive screening or follow-up prior to their cancer diagnosis. Over the 11-year surveillance period, more than a quarter of the women had some documented evidence of underinsurance in the five years prior to their invasive cancer diagnosis, with the greatest percentage of underinsurance noted among Hispanic women. We found that a history of underinsurance was independently associated with an increased likelihood of having either a “no screening” or “no follow-up” screening history compared to having progressed to cancer due to failure of the screening test. Independent of insurance history, women with a history of smoking compared to non-smokers and those with non-white race or Hispanic ethnicity were more likely to have not been screened or followed-up appropriately leading up to their invasive cervical cancer diagnosis.

Our findings are consistent with previous studies that have reported uninsured women to be less likely to have undergone screening or follow-up care in relation to cervical cancer prevention [15, 29]. Underinsured screening-eligible women among diverse populations have been shown to be less likely to be up to date with recommended screening guidelines and consequently have an increased likelihood of cancer diagnosis and presentation at a later stage of disease [3, 17, 30]. In contrast to our findings, a prior study among women who accessed preventive health services through urban community health centers (CHCs) showed uninsured women had no significant delay in receiving cervical screening and follow-up procedures or management compared to continuously insured women [31]. This difference may reflect CHCs’ provision of health services to uninsured and underserved groups and their role in reducing health services disparities [17, 32]. Studies have demonstrated increased insurance coverage among low-income populations results in increased cervical cancer screening [17, 18]. Medicaid expansion and introduction of the ACA’s increased access to private insurance markets in 2014, has been associated with increases in cervical cancer screening prevalence in both expansion and non-expansion U.S. states across CHC networks [17]. However, the greatest increases in screening rates have occurred among uninsured persons in Medicaid expansion states whereas in non-expansion states the greatest increases occurred among privately insured persons [17]. Such findings, in conjunction with ours from Davidson county in TN (a non-expansion state), suggest the potential for worsening disparities in healthcare outcomes in underinsured populations in non-expansion states, disproportionately affected by lower cervical screening rates [15]. Expanding the reach of programs such as the Tennessee Breast and Cervical Screening Program (TBCSP) may offer an avenue to bridge such gaps.

Hispanic women more frequently recorded a history of underinsurance than women in other ethnic groups in our cohort. Women of non-white race and/or Hispanic ethnicity were more likely to have a history of “no screening” or “no follow-up” compared to non-Hispanic white women in our unadjusted analyses, consistent with previous studies which have shown higher rates of underinsurance and lower screening rates in ethnic minorities and immigrant populations [6, 15, 16, 33].

Previous studies have reported similar patterns of cervical screening history among different populations diagnosed with invasive cervical cancer as was found in our study [2, 8, 34–36]. Close to two-thirds of Davidson County women had not had the recommended screening test the 5.5 years prior to cancer diagnosis, consistent with studies that have shown a history of no screening preceding most cervical cancer diagnoses [2, 8, 35]. Importantly, women enrolled in comprehensive health coverage and cervical screening programs, show very low screening non-adherence and incident cancer rates, with development of cancer mostly as a result of screening test failures [21]. Examining the screening histories of women in the context of 2012 screening guideline changes, we observed no differences in median year of cancer diagnosis across screening history categories. Screening guideline changes, informed by the natural history of cervical cancer progression, have resulted in decreased annual screening rates as well as decreases in screening rates at three- or five-year intervals [1, 13, 14]. More studies on the awareness, acceptability, and implementation of current screening guidelines among clinicians and screening-eligible populations may be needed to understand the net-effects and implications of guideline changes.

Consistent with other studies, we also found women with a history of smoking were more likely to have inadequate screening or follow-up, pointing to some of the effects of health-related behaviors on utilization of preventive health services [37].

This study has some important strengths and weaknesses to consider. We used comprehensive medical record review to investigate *all* women diagnosed with cervical cancer between 2008–2018 in a large metropolitan area in the US South, a region of the country with historically poor health outcomes and limited preventive health services. While obtaining cervical cancer screening information from medical data may be more accurate than self-report, it has inherent limitations and challenges [38]. We could not determine the screening histories of 29% of women for several reasons: limited availability of medical records for women diagnosed in the earlier years of the study period (Additional file 1: Table S3), deficits in patient recall as per provider notes, and limited access to primary care provider records Similarly, collection of data on potential barriers to screening was limited to provider documentation in the medical records—all inherent limitations to retrospective, observational investigation using medical records. Importantly, investigators made comprehensive efforts to mitigate these limitations through our thorough approach in medical records retrieval and follow-up to maximize data completeness and quality—a strength of this population-based study review. Secondly, incomplete capture of our exposure (underinsurance) and outcome (screening history) variables from medical records could result in non-differential misclassification by both variables, which could have either attenuated or exaggerated our odds ratio (OR) estimates. Also, changes in abnormal screening test follow-up guidelines over the study period may have affected screening history classification. Other study limitations included observer bias (controlled for using trained abstractors), limited generalizability as the study was confined to a single county, and too few of women with inadequate follow-up following abnormal screening to statistically examine separately.

This study shows non-adherence to recommended screening guidelines continues to precede most cases of invasive cervical cancer. Prior history of inadequate health insurance was independently associated with an increased risk of inadequate screening history among women with invasive cervical cancer. A critical examination of factors affecting screening such as insurance coverage will be key in informing interventions aimed at improving access to and receipt of cervical cancer prevention services among underscreened populations. Further, identification of implementation barriers faced by providers and among high-risk screening-eligible persons to current screening guidelines are needed to increase screening rates and reduce cervical cancer burden in the US and meet 2030 global elimination targets [32].

## Supplementary Information

**Additional file 1: Figure S1.** Screening history determination algorithm. Flowchart diagram depicting process of determining a woman’s prior cervical screening history from information found in medical records review. **Table S1.** List of immunocompromising conditions. **Table S2.** Patient characteristics by underinsurance history among Davidson County, TN, women diagnosed with invasive cervical cancer:2008-2018. **Table S3.** Patient characteristics by screening history determination status among Davidson County, TN women diagnosed with invasive cervical cancer:2008-2018.

## Data Availability

The data used in the analysis and findings presented in this manuscript were reviewed by the Centers for Disease Control and Prevention (CDC) and the Tennessee Emerging Infections Program (TN EIP). The CDC and TN EIP welcome all potential collaborators and all data access requests will be subject to a panel review.

## Abbreviations

aOR: Adjusted odds ratio
ACA: Affordable Care Act
ASCCP: American Society for Colposcopy and Cervical Pathology
CHC: Community Health Center
CIN: Cervical intraepithelial neoplasia
FIGO: International Federation of Obstetrics and Gynecology
HPV: Human papillomavirus
HPV-IMPACT: Human Papillomavirus Impact Monitoring Project
OR: Odds ratio
TBCSP: Tennessee Breast and Cervical Screening program
TN: Tennessee
US: United States
USPSTF: United States Preventive Services Task Force
