# Supplementary material for: Impact of prior underinsurance on cervical cancer screening among Davidson County, Tennessee, women diagnosed with invasive cervical cancer, 2008–2018
Source: BMC Womens Health. 2022 Mar 12;22:68. doi: 10.1186/s12905-022-01638-9 (PMC8918308; doi:10.1186/s12905-022-01638-9)
Supplement: Supplementary file 1 — Additional file 1: Figure S1. Screening history determination algorithm. Flowchart diagram depicting process of determining a woman’s prior cervical screening history from information found in medical records review. Table S1. List of immunocompromising conditions. Table S2. Patient characteristics by underinsurance history among Davidson County, TN, women diagnosed with invasive cervical cancer:2008-2018. Table S3. Patient characteristics by screening history determination status among Davidson County, TN women diagnosed with invasive cervical cancer:2008-2018. [file 12905_2022_1638_MOESM1_ESM.docx]

****Figure S1: Screening history determination algorithm**

****Case scenarios to note based on the following assumptions:***

**- If only year & month available, assume date as 15^th^ of said month**

| **Screening test date** | **Cancer diagnosis date** | **Recommended screening interval (for a Pap smear only test)** | **Decision (Screening History category)** |
| --- | --- | --- | --- |
| 12/01/2011  (day, month & year available) | 06/12/2015 | 12/12/2011 – 12/12/2014 | **Regard as screened & proceed with screening algorithm to classify into appropriate category per definitions** |
| 12/2011  (only month & year available; same month as the beginning of screening interval) | 06/12/2015 | 12/12/2011 – 12/12/2014 | **Regard as screened & proceed with screening algorithm to classify into appropriate category per definitions** |
| 11/2011  (only month & year available) | 06/12/2015 | 12/12/2011 – 12/12/2014 | **No screening**  (month & year outside recommended screening interval) |
| 2011  (only year available) | 06/12/2015 | 12/12/2011 – 12/12/2014 | **Cannot be determined** |
| 2011  (only year available) | 07/12/2014 | 01/12/2011 – 01/12/2014 | **Regard as screened & proceed with screening algorithm to classify into appropriate category per definitions**  (Can safely assume test was done in the recommended screening interval as the interval begins in January 2011) |

Supplementary Table S1— List of immunocompromising conditions

| Immunocompromising conditions |
| --- |
| AIDS or CD4 count <200/mm^3^  HIV infection  End Stage Renal Failure / Chronic Dialysis  Immunoglobulin deficiency syndromes  History of organ transplant  Current use of immunosuppressive, immunomodulator or chemotherapeutic agents (or completion within 2 weeks of invasive cervical cancer diagnosis) |

Supplementary Table S2— Patient characteristics by underinsurance history among Davidson County, TN, women diagnosed with invasive cervical cancer:2008-2018

|  | **History of underinsurance^a^** | |  |  |
| --- | --- | --- | --- | --- |
| **Characteristic** | **Yes**  **N=60** | **No**  **N=152** | **Total**  **N=212** | **p-valueǂ** |
| **Median age at diagnosis [IQR]**  **Median year of diagnosis**  **[IQR]** | 51 [44-59]  2013  [2011-2016] | 49 [39-60]  2013  [2010-2016] | 50 [40-59]  2013  [2010-2016] | 0.927  0.641 |
| **Race/Ethnicity, n (%)**  White, non-Hispanic  Black, non-Hispanic  Hispanic  Other | 33 (55)  15 (25)  11 (18)  1 (2) | 95 (62)  44 (29)  9 (6)  4 (3) | 128 (60)  59 (27)  20 (9)  5 (2) | 0.058 |
| **Symptoms at diagnosis**  Yes  No/Unknown | 56 (93)  4 (7) | 108 (71)  44 (29) | 164 (80)  37 (20) | **<0.001***** |
| **Immunocompromised**  Yes  No/Unknown | 1 (2)  59 (98) | 10 (7)  142 (93) | 11 (5)  201 (95) | 0.186 |
| **Current/Past Smoker**  Yes  No/Unknown  **Histology Type**  Squamous  Non-squamous  Unknown  **FIGO Stage**  I-IIA (local)  IIB-IV (advanced)  Unknown/Missing | 31 (52)  29 (48)  43 (72)  16 (27)  1 (2)  29 (48)  28 (47)  3 (5) | 70 (46)  82 (54)  92 (61)  58 (38)  2 (1)  90 (59)  52 (34)  10 (7) | 101 (48)  111 (52)  135 (64)  74 (35)  3 (1)  119 (56)  80 (38)  13 (6) | 0.461  0.231  0.240 |
| **Other Barriers^b^**  None  1 or more barriers | 31 (52)  29 (48) | 114 (75)  38 (25) | 145 (68)  67 (32) | **0.001**** |

**ǂ**Pearson’s chi-square / Fisher’s exact tests as applicable

**±**Wilcoxon rank-sum test

( ) Column percentages

IQR— interquartile range

**^a^**Underinsurance defined as any history of no insurance or insufficient coverage, including lapses in coverage or concerns about poor insurance coverage resulting in lack or delayed receipt of healthcare services.

^b^Other barriers includes documentation of any of the following in the five years prior to cancer diagnosis: poor English proficiency, substance use disorder, morbid obesity (body mass index ≥40 kg/m^2^), history of incarceration, history of homelessness, or serious mental illness.

Boldface p-value indicates statistical significance (*p<0.05, **p<0.01, ***p<0.001)

Supplementary Table S3— Patient characteristics by screening history determination status among Davidson County, TN women diagnosed with invasive cervical cancer:2008-2018

|  | **Screening History** | |  |  |
| --- | --- | --- | --- | --- |
|  | **Indeterminate**  **N=61** | **Determined**  **N=151** | **Total**  **N=212** | **p-value**ǂ |
| **Median age at diagnosis [IQR]**  **Median year at diagnosis**  **[IQR]**  **Race/Ethnicity**  White, non-Hispanic  Black, non-Hispanic  Hispanic  Other  **History of underinsurance^a^**  Yes  No  **Histology (Category)**  Squamous  Non-squamous  Unknown  **FIGO Stage**  I-IIA (local)  IIB-IV (advanced)  Unknown  **Symptoms at diagnosis**  Yes  No  Unknown  **Immunocompromised**  Yes  No  Unknown  **Current/Past Smoker**  History of  No/Unknown  **Other Barriers^b^**  None  1 or more barriers | 48 [40-56]  2011  [2009-2015]  34 (56)  21 (34)  5 (8)  1 (2)  13 (21)  48 (79)  41 (67)  19 (31)  1 (2)  42 (69)  11 (18)  8 (13)  47 (77)  5 (8)  9 (15)  4 (7)  40 (67)  16 (27)  30 (49)  31 (51)  47 (77)  14 (23) | 50 [41-60]  2013  [2011-2016]  94 (62)  38 (25)  15 (10)  4 (3)  47 (31)  104 (69)  94 (62)  55 (36)  3 (1)  103 (68)  43 (29)  5 (3)  117 (78)  26 (17)  8 (5)  7 (5)  135 (89)  9 (6)  71 (47)  80 (53)  98 (65)  53 (35) | 50 [40-60]  2009  [2010-2016]  128 (60)  59 (28)  20 (9)  5 (2)  60 (28)  152 (72)  135 (64)  74 (35)  3 (1)  145 (68)  54 (26)  13 (6)  164 (77)  31 (15)  17 (8)  11 (5)  175 (83)  25 (12)  101 (48)  111 (52)  145 (68)  67 (32) | 0.367  0.016  0.617  0.151  0.701  0.013  0.026  <0.001  0.776  0.085 |

ǂ Pearson’s chi-square or Fisher’s exact test as applicable for categorical variables and Wilcoxon rank-sum test for continuous variables

( ) Column percentages

IQR— interquartile range

**^a^**Underinsurance defined as any history of no insurance or insufficient coverage, including lapses in coverage or concerns about poor insurance coverage resulting in lack or delayed receipt of healthcare services.

^b^Other barriers includes documentation of any of the following in the five years prior to cancer diagnosis: poor English proficiency, substance use disorder, morbid obesity (body mass index ≥40 kg/m^2^), history of incarceration, history of homelessness, or serious mental illness.
